# Conserving threatened species during rapid environmental change: using biological responses to inform management strategies of giant clams

**DOI:** 10.1093/conphys/coab082

**Published:** 2021-12-13

**Authors:** Sue-Ann Watson, Mei Lin Neo

**Affiliations:** 1 Biodiversity and Geosciences Program, Museum of Tropical Queensland, Queensland Museum Network, 70-102 Flinders Street, Townsville, Queensland, 4810, Australia; 2 Australian Research Council Centre of Excellence for Coral Reef Studies, James Cook University, 1 James Cook Drive, Townsville, Queensland, 4811, Australia; 3 Tropical Marine Science Institute, National University of Singapore, 18 Kent Ridge Road, Singapore 119227, Singapore; 4 Department of Biological Sciences, National University of Singapore, 16 Science Drive 4, Singapore 117558, Singapore

**Keywords:** *Tridacna*, ocean acidification, light, conservation, climate change, carbon dioxide

## Abstract

Giant clams are threatened by overexploitation for human consumption, their valuable shells and the aquarium trade. Consequently, these iconic coral reef megafauna are extinct in some former areas of their range and are included in the International Union for Conservation of Nature (IUCN) Red List of Threatened Species and Convention on International Trade in Endangered Species of Wild Fauna and Flora. Now, giant clams are also threatened by rapid environmental change from both a suite of local and regional scale stressors and global change, including climate change, global warming, marine heatwaves and ocean acidification. The interplay between local- to regional-scale and global-scale drivers is likely to cause an array of lethal and sub-lethal effects on giant clams, potentially limiting their depth distribution on coral reefs and decreasing suitable habitat area within natural ranges of species. Global change stressors, pervasive both in unprotected and protected areas, threaten to diminish conservation efforts to date. International efforts urgently need to reduce carbon dioxide emissions to avoid lethal and sub-lethal effects of global change on giant clams. Meanwhile, knowledge of giant clam physiological and ecological responses to local–regional and global stressors could play a critical role in conservation strategies of these threatened species through rapid environmental change. Further work on how biological responses translate into habitat requirements as global change progresses, selective breeding for resilience, the capacity for rapid adaptive responses of the giant clam holobiont and valuing tourism potential, including recognizing giant clams as a flagship species for coral reefs, may help improve the prospects of these charismatic megafauna over the coming decades.

## Teaser

This paper explores how biological responses can inform conservation strategies of threatened giant clams through global change, including climate change, global warming, marine heatwaves and ocean acidification. Understanding responses to environmental stressors can help project future distributions of suitable giant clam habitat for protection and use in selective breeding programmes.

## Introduction

Since the onset of the Industrial Revolution about 250 years ago, human-induced global change has influenced all of Earth’s bioregions. Climate change, causing global climate destabilization, is being realized through increases in temperature, heatwaves and associated phenomena, such as ocean acidification—where carbon dioxide (CO_2_) reacts with seawater lowering the pH of the oceans. Global change has accelerated rapidly, particularly in the past 50 years, and we are tracking the worst-case emissions scenario—the business-as-usual Representative Concentration Pathway (RCP) 8.5 (Collins *et al*., 2013). This recent epoch of change is causing major ecosystem losses and a biodiversity crisis on Earth, with many species threatened or lost already in the sixth mass extinction. We are inevitably losing species before they can be described, let alone understood.

Rapid global change in the marine realm has contributed to consecutive global ocean heating events in the past decade (Bureau of Meteorology, 2020). These events have led to major coral reef bleaching around the world, including some of the world’s most pristine and protected reefs, such as the northern Great Barrier Reef (Hughes *et al.*, 2017; Hughes *et al*., 2018). Worldwide, coral reef ecosystems are in decline (IPBES, 2019); however, it is these ecosystems that play host to major groups of marine megafauna^*^ including (i) bony fishes, (ii) sea birds, (iii) giant clams, (iv) squids and octopuses, (v) sharks and rays, (vi) whales and sea cows and (vii) sea turtles (Pimiento *et al*., 2020). (^*^We note that definitions of megafauna vary among ecosystems, with size-based threshold definitions often used to define megafauna appropriate to each ecosystem (Moleón *et al*., 2020). In benthic marine ecosystems, length-based definitions are typically used for invertebrates, where megafauna can include animals such as sea stars, crabs and worms (Moleón *et al*., 2020). Here we consider giant clams as megafauna, although we acknowledge most giant clam species do not attain sizes greater than the *c*. 45 kg mass threshold (Estes *et al*., 2016) traditionally used to define animals, typically mammals (Roberts *et al*., 2001), as megafauna.)

Many of these megafaunal groups are already threatened by human overexploitation. Now global change not only threatens the coral reef ecosystems in which these megafauna live, but also directly affects megafaunal individuals through physiological responses to stressors such as elevated temperature. Giant clams, for example, contain symbiotic microalgae within their tissues, like reef-building corals do, and are thus susceptible to bleaching from elevated sea surface temperatures (SSTs) (see below). A recent analysis identified the giant clam (*Tridacna gigas*) as one of the top five marine megafauna species threatened globally, based on a functional trait and extinction risk assessment (Pimiento *et al*., 2020).

## Giant clams

**Figure 1:**
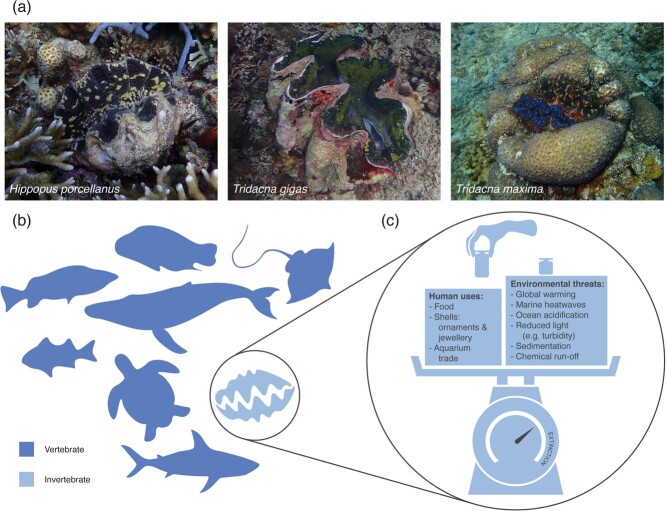
(**a**) Giant clams are highly conspicuous coral reef animals (**b**) and are considered iconic creatures in places such as the Great Barrier Reef, Australia, (**c**) but they also face increasing pressures from human overexploitation, agriculture, urbanization and global change, which may push them towards extinction.

Giant clams (Bivalvia: Cardiidae: Tridacninae) are large marine bivalves inhabiting coral reefs across the Indo-Pacific, with 12 recognized species in two genera (*Hippopus* and *Tridacna*) (Tan *et al.*, 2021; Fig. 1a). The largest of them all is *T. gigas* (also known as the true giant clam), a species that can weigh over 250 kg and measure over 1.3-m long (Rosewater, 1965), producing the biggest shell in the world. These charismatic bivalves are also characterized by their longevity (i.e. predicted maximum life span of 100 years), late reproductive maturity (i.e. 7–10 years for females), mostly sessile habit after settlement and dependence on photosynthesis (Yamaguchi, 1977; Lucas, 1994; Ungvari *et al.*, 2013).

Within coral reef ecosystems, giant clams are known to make important contributions such as enhancing the net primary productivity of coral reefs by mixotrophy, nutrient recycling, provisioning of nurseries and shelters for other reef animals and serving as reservoirs of viable symbionts (Neo *et al.*, 2015; Umeki *et al.*, 2020). Giant clam calcification also contributes to carbonate budgets and is estimated to contribute 0.7–9.0% of the mean calcium carbonate budget of Red Sea coral reef communities (Rossbach *et al*., 2021). The provision of structural refugia in particular scores bivalves, including giant clams, highly for their functional importance on coral reefs (Wolfe *et al.*, 2020). Worldwide, the giant clam (*T. gigas*) is one of the top three functionally unique marine megafauna species, along with the dugong and green sea turtle (Pimiento *et al*., 2020).

Giant clams are also significant coastal resources for humans and have been consumed for their meat for millennia. In Pacific Island countries and territories, giant clams are the main invertebrate harvested (~28% catch) by gleaning and free diving (Bell *et al.*, 2011). Giant clams are also used as materials for their strong calcified shells (Lyons *et al.*, 2018), and more recently as reef pets in the ornamental aquarium trade (Mies *et al.*, 2017). Giant clams are recognized as iconic creatures in coral reef tourism and feature as the only invertebrate among the eight iconic creatures of the Great Barrier Reef, Australia (Barrier Reef Australia, 2021) (Fig. 1b).

## ‘Solar-powered’ animals: benefits and vulnerability

Multicellular animals that have captured single-celled algae within their tissues include corals and giant clams. This ‘solar-power’ capability arises from photosynthesis by tiny algal protists—endosymbiotic dinoflagellates from the family Symbiodiniaceae, also known as zooxanthellae—within their bodies, providing the animal host with an additional energy source other than the ingestion of food items.

Giant clams can thus obtain nutrients via two pathways: photosynthesis and filter-feeding. These mixotrophic bivalves depend heavily on their endosymbionts to acquire the bulk of their carbon and nitrogen requirements for growth and metabolism (Klumpp and Griffiths, 1994; Hawkins and Klumpp, 1995), even though they possess the functional gills and digestive systems typical of heterotrophic bivalves. In giant clams, the reliance on phototrophy increases with body size. For example, small *T*. *gigas* individuals (shell length, ~43 mm) obtain 65% of their carbon from filter-feeding, compared to 34% for larger individuals (shell length, ~167 mm) (Klumpp *et al.*, 1992), and this trend continues as individuals grow (e.g. Klumpp and Griffiths, 1994). Indeed, at normal depths and light levels, phototrophy alone may provide most, if not all, of the carbon requirements in giant clam species (*T*. *gigas*: Fisher *et al*., 1985; Klumpp *et al*., 1992; *T. mbalavuana* and *T. derasa*: Klumpp and Lucas, 1994), and *T. squamosa* juveniles can survive for 10+ months with light as the sole energy source (Fitt and Trench, 1981). The combination of phototrophy and heterotrophy is thought to explain why giant clams have rapid growth rates and likely allows them to grow to large body sizes compared to other bivalves (Klumpp and Griffiths, 1994). However, this heavy reliance on phototrophy to meet their energy needs, especially with increasing body size, makes giant clams more sensitive and thus vulnerable to increased SSTs caused by global warming (e.g. Andréfouët *et al.*, 2013; Van Wynsberge and Andréfouët, 2017; Van Wynsberge *et al.*, 2018).

At local and regional scales, giant clams face other human-induced pressures such as overfishing, habitat degradation and coastal urbanization (Neo *et al.*, 2017; Neo, 2020; Fig. 1c), which reduce densities of populations across their ranges in the wild. Consequently, stock depletion impedes their reproductive success in nature as giant clams rely on synchronized broadcast spawning among conspecific individuals (Lucas, 1988). As these threats still persist today, the number of viable spawning individuals is rapidly dwindling and populations face imminent declines due to poor reproductive success (Gwyther and Munro, 1981).

## ‘Traditional’ conservation solutions

Giant clams are protected species under Appendix II of the Convention on International Trade in Endangered Species of Wild Fauna and Flora (CITES), which consists of species that are not necessarily currently threatened with extinction but may become so unless trade is closely controlled. Of the 12 recognized giant clam species, only 9 have been assessed and listed in the International Union for Conservation of Nature (IUCN) Red List of Threatened Species: *T*. *gigas*, *T*. *derasa*, *T. rosewateri* and *T*. *mbalavuana* are listed as Vulnerable and *Hippopus hippopus*, *H. porcellanus*, *T. maxima*, *T*. *squamosa* and *Tridacna crocea* are listed as Lower Risk (Wells *et al.*, 1983; Wells, 1997). At local and regional scales, several countries have specific laws to protect their giant clam stocks (see Neo, 2020). While these measures are in-place to safeguard these threatened bivalves from overexploitation, there are several shortcomings. Due diligence on the enforcement of CITES is largely dependent on whether trading countries are signatories to the CITES Treaty, and there is an urgent need to update the IUCN Red Listings to reflect the contemporary status of each giant clam species (Neo, 2020). For example, the recently rediscovered species (*T. squamosina*, *T. noae* and *T. elongatissima*) have yet to be assessed, and due to lack of current knowledge about their biology and abundance, they will likely be classified as ‘Data deficient’.

Breeding programmes have existed since the late 1980s to help restock over-exploited wild populations of giant clams (e.g. Gomez and Mingoa-Licuanan, 2006; Moorhead, 2018). Gametes are collected from broodstock and subsequent larvae reared in laboratory-style facilities with juveniles grown out in land- and/or ocean-based aquaculture settings, both land and ocean based. This mariculture (seawater aquaculture) of giant clams has been a feasible technique to mass produce individuals for restocking rare species or extirpated populations in several regions, such as the Pacific Islands, Southeast Asia and Japan (e.g. Heslinga *et al.*, 1984; Gomez and Mingoa-Licuanan, 2006; Kurihara *et al*., 2012; Neo and Todd, 2012). Despite the extensive application of mariculture and restocking in numerous countries, the rates of success of these initiatives are neither well studied nor well documented (Teitelbaum and Friedman, 2008; Moorhead, 2018). Some of the possible challenges that clam breeders face are operations related such as recurring high mortality rates, coupled with high running costs and labour-intensive rearing. Also, hatchery-bred clams, spawned from an inherently sub-sampled population (broodstock), are likely to be less genetically diverse (Benzie and Williams, 1996), which could increase their vulnerability to diseases and/or environmental change stressors.

## New conservation considerations in a rapidly changing world

In recent years, we are recognizing that giant clams are not only threatened by overexploitation by humans, but are also threatened by a suite of environmental change drivers including (i) global change stressors, such as climate change, global warming and ocean acidification, and (ii) local and regional stressors from agriculture and urbanization, such as turbidity and sedimentation. Of the global pressures on marine life, pollution and climate change continue to occur at peak pressures (Duarte *et al*., 2020). Crucially, the effects of local to global environmental stressors not only act in isolation, but also in concert, with potentially unknown synergistic effects occurring within the complex coastal environments that giant clams inhabit.

### Global-scale environmental stressors

#### Ocean warming

During the past 40 years, the Indo-Pacific warm pool, where SSTs are permanently over 28°C, has expanded nearly two-fold in area covering much of the Indo-Pacific region (Roxy *et al.*, 2019) and affecting large areas of the range of giant clams. The highest temperatures occur within the centre of giant clam diversity in an area around the Coral Triangle—a region considered the global centre of marine biodiversity in the Indonesian-Philippines and Far Southwestern Pacific bioregions.

**Figure 2:**
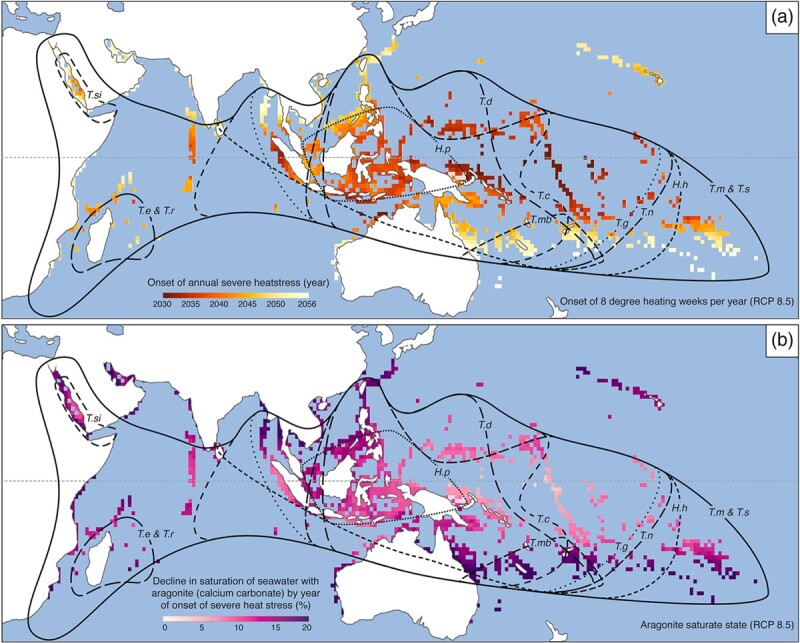
(**a**) Projected heat stress caused by global warming and (**b**) reduced saturation state of seawater with respect to aragonite (caused by ocean acidification) will affect areas within the ranges of all giant clam species. Abbreviations for giant clam species: *T*.*si*, *T. squamosina*; *T*.*e*, *T. elongatissima*; *T*.*r*, *T. rosewateri*; *H*.*p*, *H. porcellanus*; *T*.*d*, *T. derasa*; *T*.*mb*, *T. mbalavuana*; *T*.*c*, *T. crocea*; *T*.*g*, *T. gigas*; *T*.*n*, *T. noae*; *H*.*h*, *H. hippopus*; *T*.*m*, *T. maxima*; *T*.*s*, *T. squamosa*. Base map data from van Hooidonk *et al*. (2014) reproduced with permission from Ruben van Hooidonk (National Oceanic and Atmospheric Administration Coral Reef Conservation Program and the University of Miami).

Heat stress in the marine environment is generally measured by SST metrics, such as degree heating weeks (DHW, where SST anomalies over the 1985–1993 baseline are summed). DHW are often used to indicate the likelihood of coral bleaching (although see McClanahan *et al*., 2019 for a comparison of conventional and new SST metrics). Global heat stress since the year 2015 has been substantial, affecting large areas of the Indo-Pacific. Further projected heat stress from continued global warming will affect large areas of the range of all giant clam species (Fig. 2a). In Fig. 2a, projected marine heat stress is indicated by the onset of 8 DHW per year under RCP8.5. We acknowledge that although SST metrics, such as DHW, are generally used for corals, they could act as proxies for bleaching in giant clams, and the development and use of heat stress metrics for giant clams is an area worth future research.

Elevated temperatures cause a range of physiological effects in giant clams including decreases in photosynthetic activity, changes in respiration rate (Blidberg *et al.*, 2000) and effects on symbiont photosynthetic yield and density (Brahmi *et al.*, 2019) and enzyme activities (Zhou *et al.*, 2019). Like corals, giant clams bleach in response to high temperatures and high light intensities (Buck *et al.*, 2002), with documented cases of bleaching in giant clams in the wild from high temperatures (e.g. Addessi, 2001; Andréfouët *et al.*, 2018; Apte *et al.*, 2019). Elevated seawater temperature also changes fatty acid composition in *T*. *maxima* (Dubousquet *et al.*, 2016), affects embryonic and larval development (in *T*. *gigas*) (Enricuso *et al.*, 2019) and leads to oxidative stress and collapse of the clam-algae symbiosis (in *T*. *crocea*) (Zhou *et al.*, 2019). A review of French Polynesian giant clam populations concluded that abnormal weather conditions linked to climate change, climate anomalies and global warming occurred in all documented cases of mass mortality of giant clams (Van Wynsberge and Andréfouët, 2017), highlighting the dominance of temperature as the primary environmental stressor involved in giant clam mass mortalities.

#### Ocean acidification

CO_2_ from the atmosphere is absorbed by the oceans where it reacts with seawater and lowers pH in a process called ocean acidification. This process has caused a decline in the saturation state of seawater with respect to calcium carbonate polymorphs including aragonite (Jiang *et al*., 2015)—a more soluble polymorph compared to calcite and the main form of calcium carbonate in giant clam shells (Moir, 1990). Marine animals that produce calcium carbonate shells and exoskeletons are thus particularly vulnerable to ocean acidification. For giant clams, which produce such large shells and are thus very heavily calcified, ocean acidification could be particularly problematic. Projected continued reductions in aragonite saturation state will be experienced by all giant clam species across their ranges (Fig. 2b). Lower seawater saturation state occurs in an opposite latitudinal gradient to ocean warming, meaning there is no safe haven for coral reef species at low or high latitudes from the combined impacts of warming and acidification (van Hooidonk *et al*., 2014).

In giant clams, ocean acidification leads to reduced survival (Watson *et al*. 2012a; Watson, 2015) and growth (Watson, 2015; Kurihara and Shikota, 2018; Brahmi *et al.*, 2019). The effect of ocean acidification on giant clams can also depend on temperature (Watson *et al*., 2012a) and light availability (Watson, 2015) demonstrating interactions among stressors in the marine environment.

For animals with symbiotic microalgae, increased CO_2_ availability could lead to enhanced primary production of energy. However, although elevated CO_2_ increased endosymbiont density in the giant clam *T. crocea*, endosymbiont productivity did not change, which suggests productivity per endosymbiont decreased at elevated CO_2_, and thus negative effects of CO_2_ were not countered by any potential increases in photosynthesis (Kurihara and Shikota, 2018). Ocean acidification also leads to altered behaviour in invertebrates (Manriquez *et al.*, 2013; Watson *et al.*, 2014), including tropical molluscs (Watson *et al*., 2014, Watson *et al*., 2017a, Spady *et al*., 2014; Spady *et al*., 2018; Thomas *et al*., 2021) likely through mechanisms including disrupted functioning of ligand-gated chloride channels, such as GABA_A_ receptors (Watson *et al*., 2014, Thomas *et al*., 2020; Thomas *et al*., 2021), further increasing the vulnerability of calcareous taxa. Behavioural changes could affect settlement choices in invertebrate planktonic larval stages and antipredator responses in planktonic and settled life stages.

#### Ocean deoxygenation

Oxygen loss in the ocean, known as ocean deoxygenation or hypoxia, is emerging as a pervasive negative threat to marine life across multiple taxonomic groups (Sampaio *et al*., 2021; Sutherland *et al*., 2021). Deoxygenation is likely an increasingly important, but underestimated and underreported, source of mortality on coral reefs (Altieri *et al*., 2017; Breitburg *et al*., 2018). While the giant clam holobiont might produce a net increase in oxygen during the day, night time respiration requires a net use of oxygen, and it is thus during dark periods that giant clams are most likely to be susceptible to hypoxic conditions. Overall, in *T*. *squamosa*, oxygen uptake exceeds total oxygen production and oxygen extraction in the dark is high relative to species without photosymbionts (Mangum and Johansen, 1982), suggesting that giant clams could be susceptible to ocean deoxygenation.

#### Salinity change

Ocean salinity is also changing as a response to global warming with higher salinity regions becoming more saline and fresher regions becoming less saline (Durack *et al.*, 2012). Studies show some giant clam species are tolerant to hyposaline conditions (*T*. *squamosa*: Neo *et al.*, 2013; Eckman *et al.*, 2014), whereas others are affected by low salinities (*T*. *gigas*: Maboloc *et al.*, 2014; Maboloc *et al.*, 2015; Sayco *et al.*, 2019) or low and high salinities (*H. hippopus*: Panggabean *et al.*, 2009). Overall, since giant clams have some tolerance to a range of salinities (e.g. Maboloc and Villanueva, 2017), the small scale changes currently occurring in ocean salinity, within the order of ±0.1 or 0.2 units (Durack *et al.*, 2012), are unlikely to be a direct stressor compared to other global changes.

### Local- to regional-scale environmental stressors

#### Light availability

A range of local- to regional-scale coastal activities, such as terrestrial run-off of sediments and chemicals from agriculture and urban areas, can lead to a reduction in light availability (often measured by photosynthetically active radiation, PAR) in the water column, known as coastal ocean darkening. Since light regimes structure aquatic food webs, this water column light attenuation could be a key driver in coastal food web changes (Aksnes *et al*., 2009).

‘Solar-powered’ animals with symbiotic microalgae within their bodies necessarily have a strong interdependence on optimum light levels in their environment. Giant clams have a range of light levels in which they can survive and within that, optimum light levels for growth. For instance, studies have found that *T*. *squamosa* can engage in light-enhanced growth and shell formation through the increased expression of specific proteins to facilitate transport of inorganic nutrients from the clam host to its endosymbionts (Ip *et al.*, 2017; Chew *et al.*, 2019).

In giant clams, calcification and primary production are dependent on light and are highest at incident light levels equivalent to 3–5 m water depths (Rossbach *et al*., 2019a). On the other hand, too much light can induce changes in chlorophyll content or alter endosymbiont cell size and populations and can cause bleaching (Buck *et al.*, 2002). At the other end of the scale, too little light is problematic with low light levels reducing giant clam survival (Watson, 2015; Eckman *et al.*, 2019). The optimum light range is likely to differ among species, with some species such as *T*. *crocea* often found in very shallow water, and other species like *T*. *mbalavuana* found up to 30-m deep (Neo *et al.*, 2017). Indeed, different depth distributions among species may be explained by their degree of mixotrophy. In the Red Sea for example, *T*. *maxima* is a strict functional photoautotroph and exhibits a shallow depth distribution (down to ~10 m, maximum 17 m), whereas *T*. *squamosa* extends its depth range (down to 42 m) by heterotrophy (Jantzen *et al*., 2008).

Humans have altered the flux of sediment reaching the global coastal oceans (Syvitski *et al*., 2005). Processes such as turbidity and sedimentation both reduce light availability and create physical disturbance when particles settle onto the mantle (photosynthetic upward facing soft tissues) of the clam. Increased seawater sediment loading results in more mantle contractions (Elfwing *et al.*, 2001), presumably to rid the mantle of sediment; however, this activity would dramatically increase demand on the energy budget of otherwise relatively inactive giant clams. Since clams from disturbed sites with higher turbidity and nutrient loading have reduced photosynthetic activity (Elfwing *et al.*, 2003), their ability to recoup energy stores is likely to be diminished.

#### Other factors

Other urbanization factors, such as pollution, also affect giant clams. A study on *T*. *squamosa* found that increased heavy metals (copper) in seawater decreased gross production:respiration ratio by about one third through decreased photosynthesis (Elfwing *et al.*, 2001). Tourists too have the potential to affect giant clams. The presence of movement or dark silhouettes above giant clams leads to mantle contractions and/or shell contractions as a natural anti-predator response. Thus, snorkelers and divers can cause partial or full shell closure by swimming above and around clams, touching clams and by kicking up sediment from the seafloor that could land on the clam’s mantle. Tourism activities may also generate pollution, including from boats (chemical and noise pollution) and marine litter.

While these above factors tend to act on local to regional scales, they will almost certainly interact with stressors occurring on global scales, such as global warming and ocean acidification. The potential synergistic effects of multiple stressors and interactions in the natural environment remain little studied, in part because of the complexity; however, experiments investigating multiple broad-scale stressors suggest that combined stressors can result in particularly negative effects on giant clams with increased lethal and sub-lethal effects (T. squamosa Watson *et al.*, 2012a; Watson, 2015; although compare T. maxima Brahmi *et al.*, 2019; Armstrong *et al.*, 2020).

## Responses to environmental change

The responses of giant clams to environmental change may include the following: shifts in biogeographical and/or depth distributions (mid- to longer-term); changes in behaviour and/or physiology, including modifying symbiont communities (short- to mid-term), phenotypic plasticity (mid-term), genetic adaption (longer-term); and extinction. Distribution shifts can occur in response to a changing environmental condition that occurs along a gradient (e.g. temperature with latitude and light with depth). After considering (i) distribution shifts, we explore (ii) the capacity for rapid adaptive responses to environmental change from fast to slower timeframes.

### Distribution shifts

#### Biogeographical distribution—reduced latitudinal habitat availability

As global temperatures warm with climate change, species can take advantage of the decreasing planetary temperature gradient from equatorial to polar latitudes and shift their distribution poleward towards higher latitudes and cooler conditions (Lambers, 2015). Global warming has already caused a poleward range expansion of many terrestrial and marine species (e.g. Pecl *et al*., 2017). Giant clams produce pelagic larvae that are dispersed by ocean currents indicating that range expansion of giant clam species as ocean temperatures warm is possible. Range expansions of corals (Yamano *et al*., 2011) and coral reef-associated fishes (Booth *et al*., 2018) have already been observed. Most giant clam species do not need corals to survive, although wild *T. crocea*, the boring clam, bores into large boulder-shaped coral colonies or other calcium carbonate-based structures.

However, the saturation state of seawater with respect to calcium carbonate polymorphs such as aragonite decreases with increasing latitude. Light availability, a key metric for the success of giant clams, also decreases with latitude. Thus, the formation and persistence of giant clam shells (the production of which may represent a greater proportional cost to the total energy budget than the shells of less calcified molluscs; see Watson *et al*., 2012b; Watson *et al*., 2017b) and the ability of giant clam photosymbionts to capture light are both likely to diminish on a poleward trajectory. Consequently, a poleward shift may well be limited and overall likely to result in a reduction in available latitudinal habitat size primarily driven by a range retraction from lower latitudes. In some locations, such as the Red Sea, giant clams also face a physical poleward migration barrier. This puts species in these locations at greater risk from global change. These factors, and further critical unknowns, such as local water quality conditions and interactions with other local- to global-scale environmental drivers, mean a reliance on a poleward distribution shift for the persistence of giant clam species is likely a risky strategy. Furthermore, in sedentary animals, such as giant clams, distribution shifts will tend to occur over multiple generations and may well not keep pace over ground with global change.

#### Depth distribution—reduced habitat availability within a species’ depth range

**Figure 3:**
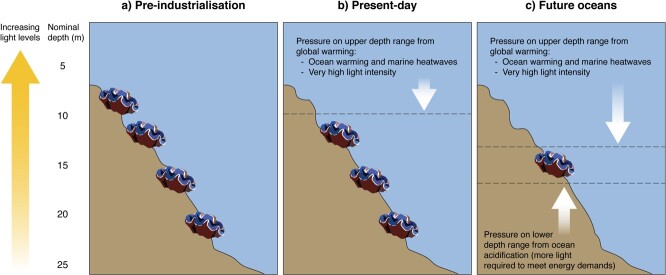
(**a**) Giant clam depth distributions are influenced by environmental conditions and are likely to be limited by (**b**) current and (**c**) continued global change. Increasing CO_2_ levels cause ocean warming and acidification, which are likely to compress the upper and lower limits, respectively, of the depth distributions of giant clams, especially in combination with other factors such as reduced light availability. Giant clam symbol credit: Tracey Saxby, Integration and Application Network (ian.umces.edu/media-library).

Physiological responses of giant clams to global- and local–regional-scale stressors may influence their ecology on coral reefs, for example by altering their natural depth distribution. High SSTs and high light intensities from solar irradiation limit the upper depth distribution of giant clams (Fig. 3a). Uppermost depth distributions will move deeper because high temperature and intense light levels will bleach giant clams or cause other physiological stress responses (Fig. 3b). The lower depth distribution of giant clams is dictated by low light levels. Reduced light levels at the deepest depth distribution through natural light attenuation in seawater, especially in combination with other stressors such as elevated temperatures and ocean acidification that cause lethal and sub-lethal effects at low light (Watson *et al.*, 2012a; Watson, 2015), are likely to create a shoaling effect, limiting the lower depth distribution of photosymbiotic giant clams (Fig. 3c). Increased turbidity or sediment load will further reduce light availability and thus reduce habitable depth even more. Compressed depth distributions with global change will limit giant clam habitat on reefs leading to an overall reduction in suitable habitat within their natural range.

Once giant clams recruit to the coral reef substrata, they are essentially sessile, like reef-building corals, and cannot move to find more optimal habitat should environmental conditions change. Although juvenile clams have some ability to move with their foot once they settle onto the coral reef (Soo and Todd, 2014), this movement is limited and they will soon attach themselves permanently with byssal threads or bore into the substrata (in the case of boring species). Older, and thus, larger giant clams become too heavy and are unable to change their position on reefs. Given limited or no ability to move, giant clams need stable local conditions, such as light availability through water clarity, to persist.

Modelling of available habitats using present-day and projected future physical and chemical parameters (such as depth, temperature, aragonite saturation state and light levels) could be used to estimate the effect of reduced depth distributions on the total habitat availability within a species range. This modelling approach could be used to investigate the vulnerabilities of each giant clam species since they have different depth distributions, with certain species, such as *T*. *mbalavuana*, presumably able to tolerate lower light levels naturally.

Considering depth distribution in giant clams provides an example of how physiological responses may help predict ecological patterns and inform conservation management approaches of these threatened species. The principles of this approach, as well as other aspects of the current paper, could also be applied to other sessile photosymbiotic animals, such as reef-building corals.

### Capacity for rapid adaptive responses

#### Behaviour

Although sessile beyond juvenile stages, giant clams have the ability to open and close their shell using their large adductor muscle. This immediate behavioural response to changing conditions is one way that clams can respond to environmental change (Dehaudt *et al.*, 2019) and in a different way compared to corals. This distinct physiological advantage allows them to expose or protect their symbiotic tissues from light and predators. In low daylight conditions, giant clams can extend their mantle to maximize light capture. At night, they retract their mantle and partially close their shells, presumably to deter predators, and because there is no light for photosynthesis. During periods of elevated temperatures or very high light levels, giant clams have the option to partially close their shells and retract their mantle, providing shading protection for themselves and their symbionts (Rossbach *et al*., 2020).

#### Microorganisms associated with the giant clam holobiont

Like corals, giant clams are holobionts, harbouring a range of microorganisms within their tissues, such as algae (endosymbiotic dinoflagellates), bacteria (Rossbach *et al.*, 2019b; Guibert *et al*., 2020) and, potentially, fungi and viruses as well. Associations of the host with microorganisms offer the potential for rapid adaptive responses to environmental change in corals (Torda *et al*., 2017), and similarly in giant clams.

While the stony corals hold their symbiotic microalgae in the endodermal cells lining the gastrovascular cavity, the microalgae endosymbionts in giant clams are found intercellularly within the siphonal mantle, specifically in the tertiary tubes of the zooxanthellal tubular system (Norton *et al.*, 1992). This location of symbionts could offer increased protection against rapid environmental changes and possibly confer some resistance to bleaching, compared with corals. There have been instances during bleaching events where corals have either bleached or died due to heat stress but giant clams have not (Neo *et al.*, 2017).

Studies have also found that microalgae endosymbiont distribution and diversity in giant clams can be driven by local conditions such as ambient temperature levels and temperature fluctuations (Lim *et al.*, 2019). During the ontogeny of giant clams from juveniles to adults, the composition and densities of endosymbionts may shift, often also as a result of changing environmental conditions (Belda-Baillie *et al.*, 1999). In addition, the diversity and community structure of Symbiodiniaceae in giant clams likely affects host traits such as growth rate, reproduction and photosynthetic efficiency (DeBoer *et al.*, 2012). Giant clams mostly associate with Symbiodiniaceae from the genera *Symbiodinium* (clade A), *Cladocopium* (clade C) and *Durusdinium* (clade D) (Ikeda *et al.*, 2017; Lim *et al.*, 2019). Hosting multiple endosymbiont species likely enables giant clams to cope better with changing environments, as each endosymbiont species has different tolerances to temperature, irradiance and turbidity. Within the host, endosymbiont communities are not static and host-endosymbiont shuffling can occur as the holobiont acclimatizes to changing environmental conditions (Belda-Baillie *et al.*, 1999).

Recently, different microbial communities, or ‘microbiotypes’, have been found associated with giant clams; three microbiotypes were found in a study of *T. maxima* individuals from French Polynesia (Guibert *et al.*, 2020). Seawater temperature and the presence of different corals did not change the composition of Symbiodiniaceae or bacterial communities, but the giant clam biotype dominated by the bacterial family Vibrionaceae was linked to increased host mortality, especially in the presence of the coral *Acropora cytherea*, and this effect on mortality was amplified at elevated temperatures (Guibert *et al.*, 2020).

#### Phenotypic plasticity and genetic adaptation

The phenotype of an organism can be modified through nongenetic processes, known as phenotypic plasticity, acclimation or acclimatization. Nongenetic processes can occur within a generation (e.g. reversible or developmental acclimation) and between generations (e.g. transgenerational acclimation) through parental provisioning, hormones and proteins and epigenetic marks (Munday, 2014). Genetic adaptation occurs between generations when chance genetic mutations produce favourable modifications. While both these processes are likely to occur in giant clams (e.g. Neo and Todd, 2011), characteristics of giant clam life history traits mean they will play out over longer timescales in comparison to many other coral reef organisms.

Giant clams are long-lived, and larvae that are produced in present-day oceans could survive until the end of the century. However, the opportunity for early life developmental acclimation in individuals will be limited to present-day conditions. Additionally, giant clams take a long time to reach reproductive maturity. They produce first male, then male and female gametes, when they reach a larger size. This means giant clam generation times are long, and reduced generation turnover time limits the potential for genetic adaption. Additionally, the selective exploitation of large giant clams from the wild will reduce the proportion of female clams. On the other hand, one positive characteristic of giant clam biology is that they can spawn millions of gametes, potentially producing high numbers of larvae and thus increasing the chances of selection acting to produce fitter individuals.

## Conservation strategies through rapid environmental change

In addition to traditional breeding programmes, we should now view the conservation of giant clams through a global change lens to focus on protecting wild populations of these threatened species in the face of rapid environmental change. Conservation strategies and actions include (i) the management of local and regional conditions close to optimal while global CO_2_ emissions are stabilized, (ii) adaptive population management, (iii) selective breeding of tolerant giant clam holobionts for restocking programmes in selected areas and (iv) inoculation of tolerant strains of microalgae endosymbionts in giant clam early life stages.

### Management of local and regional conditions while global CO_2_ emissions are stabilized

**Figure 4:**
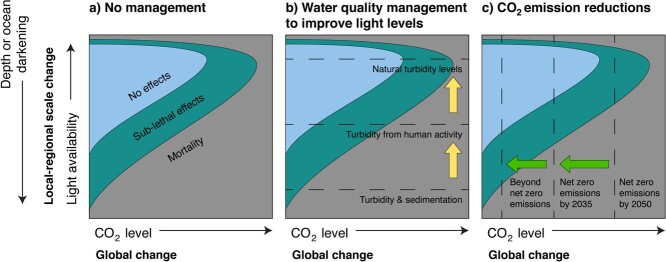
Conservation strategy conceptual model for giant clams. (**a**) Local–regional- and global-scale stressors deviate from optimum conditions (no effects) for giant clams and cause a range of sub-lethal and lethal effects. (**b**) Managing for water quality on local–regional scales can ameliorate negative effects caused by low light levels. (**c**) Rapidly reducing global CO_2_ emissions will help ameliorate the lethal and sub-lethal impacts of elevated temperatures and ocean acidification.

While management of global change stressors involves moving to net zero CO_2_ emissions as soon as possible, and potentially implementing CO_2_ capture technologies, management of local–regional-scale stressors will involve water catchment management to maintain or improve water quality. Managing terrestrial run-off into the marine environment in areas of giant clam habitats will ensure associated impacts do not add further stress to giant clams. Additional impacts from stressors, such as reduced light levels (Watson, 2015), can push the response of giant clams to elevated temperature and ocean acidification from sub-lethal to lethal effects (Fig. 4a). Maintaining giant clam habitats in the stable ‘no effects’ zone through management of local–regional-scale (Fig. 4b) and global-scale (Fig. 4c) stressors is key to their persistence.

### Adaptive population management

Conservation actions for giant clams during rapid global environmental change are not necessarily straightforward and may involve ‘aquaculture-style’ or ‘higher-tech’ solutions including some of those outlined in the sections below. However, a range of ‘lower-tech’ conservation actions have been adopted over the past 10–20 years, or are being considered, in the face of rapid environmental change, and focus on the management of wild populations of giant clams. Broadly, these conservation actions focus on addressing the local challenges directly such as restocking and/or reintroducing cultured giant clam species to sites where they are severely depleted or extirpated due to overexploitation, increasing frequency of monitoring and surveillance of wild stocks after bleaching events, translocating individuals to cooler sites or latitudes to avoid high seawater temperatures and enhancing the socioeconomic value of giant clams through tourism (see Table 1 for details).

**Table 1:** Threats and conservation actions

Region	Species diversity	Current threats	Conservation actions
Red Sea and Gulf of Aden	Ts, Tm, Tsi	• Global warming • Ocean acidification • Deoxygenation? • Degraded habitats • Water pollution (e.g. sewage discharges) • Unsustainable tourism • Coastal development	• Attempts to cultivate giant clams were carried out in early 2000s (Roa-Quaoit, 2005) • Collecting wild giant clams in Saudi Arabia has been banned since early 2000s (AbuZinada *et al.*, 2004)
Western Indian Ocean	Tc, Ts, Tm, Tr, Te	• Global warming • Ocean acidification • Deoxygenation? • Overfishing • Ornamental shell trade	• Considerations to protect and aggregate remaining wild adults to facilitate spawning, breeding and releasing hatchery-reared clams in the Republic of Mauritius (Ramah *et al.*, 2019) • Considerations to leverage on mariculture for restocking/reintroduction to prevent further depletion of stocks in Lakshadweep (Apte *et al.*, 2019)
Bay of Bengal and Andaman	Hh, Tg, Tc, Ts, Tm	• Global warming • Ocean acidification • Deoxygenation? • Overfishing • Sedimentation	• Restocking and/or reintroducing of giant clam species to sites where they have been extirpated (Teitelbaum and Friedman, 2008)
South China Sea	Hh, Tg, Td, Tno, Tc, Ts, Tm	• Global warming • Ocean acidification • Deoxygenation? • Ornamental shell trade (ivory of the sea) • Overfishing	• Selective crossbreeding individuals from geographically distinct areas to increase robustness (Zhang *et al.*, 2020) • Restocking and/or reintroducing of giant clam species to sites where they have been extirpated (Gomez, 2015)
Coral Triangle	Hh, Hp, Tg, Td, Tno, Tc, Ts, Tm	• Global warming • Ocean acidification • Deoxygenation? • Overfishing • Illegal harvesting • Aquarium trade • Degraded habitats • Water pollution • Urbanization of coastal areas	• Giant clam gardens in Samal and Tawi-Tawi, the Philippines for tourism • Restocking and/or reintroducing of giant clam species to sites where they have been extirpated (Teitelbaum and Friedman, 2008)
Australia	Hh, Tg, Tmb, Td, Tno, Tc, Ts, Tm	• Global warming • Ocean acidification • Deoxygenation? • Poor water quality (from sediments, nutrients and contaminants)	• Snorkel trails at Magnetic Island for tourism (pers. comms., R. Braley)
Pacific Ocean	Hh, Tg, Tmb, Td, Tno, Tc, Ts, Tm	• Global warming • Ocean acidification • Deoxygenation? • Overfishing • Illegal harvesting	• Restocking and/or reintroducing of giant clam species to sites where they have been extirpated (Teitelbaum and Friedman, 2008; Moorhead, 2018) • Translocating individuals to cooler sites or provisioning of shade structures during periods of high seawater temperatures (Andréfouët *et al.*, 2013) • Placing strict measures such as banning clam fishing for commercial use, setting minimum size limits for subsistence harvesting, imposing harvesting quotas or bag limits, restricting clam fishing to free diving only, banning use of mechanical fishing equipment (Andréfouët *et al.*, 2013; Kinch and Teitelbaum, 2010)

*Hh*, *H. hippopus*; Hp, *H. porcellanus*; Tg, *T. gigas*; Tmb, *T. mbalavuana*; Td, *T. derasa*; Tno, *T. noae*; Tc, *T. crocea*; Ts, *T. squamosa*; Tm, *T. maxima*; Tr, *T. rosewateri*; Tsi, *T. squamosina*; Te, *T. elongatissima*

Giant clam populations can also be managed in high-traffic tourist areas. Although tourists have the potential to contribute some local-scale disturbances to certain giant clam individuals, such highly localized impacts are likely to be easily managed and, importantly, the value of tourists seeing and appreciating giant clams in nature will likely outweigh the minimal impacts of tourist operations (see Tourism potential section below). Management of giant clams in such areas will involve awareness that clams may be using more energy to cope with some physical disturbance from tourist operations. Although supplemental feeding of giant clams is applied in aquarium settings, management of increased energy usage by giant clam individuals in the ocean could involve protecting them from other stressors such as high temperatures and subsequent bleaching, particularly since they may already be under energy stress. Management actions such as the provisioning of shade structures could also be applied to help protect specific giant clam individuals during periods of high seawater temperatures.

### Selective breeding of tolerant holobionts for restocking programmes

Aquaculture breeding programmes often select for the best performing individuals, such as for growth or disease resistance. A recent mariculture study on *T*. *crocea* found that reciprocal hybrids produced from crossbreeding individuals from two geographical populations, separated by ~600 km in distance, were more robust in terms of growth and survival than pure populations (i.e. breeding different individuals within the same geographic population) (Zhang *et al.*, 2020). Giant clam aquaculture could therefore select for tolerant individuals for current conditions and/or by introducing stressor conditions. Broodstock or offspring could also be acclimated/grown at ‘moderate’ climate scenarios (e.g. an overlaid seawater temperature of 1°C higher than ambient) to increase the resilience of individuals, or promote adaptation of the giant clam holobiont as a whole. Elevated temperature conditions could be achieved by active heating with heaters controlled by thermostats, or passive heating, for example, by using a greenhouse (Braley *et al*., 1992) or by the strategic removal or reduction of shade cloth coverings over areas of tank water. Shade cloth can also be used to establish shading regimes equivalent to particular seawater depths (e.g. 83% light = 1 m depth to 11% light = 28 m depth; Klumpp and Lucas, 1994), and this could promote resistance to higher or lower light levels (e.g. with turbidity) within the water column. Selective breeding is particularly amenable in giant clams, where spawning can produce many millions of larvae, so there is great potential for selection to new environmental conditions during early life. This process could produce individuals that have experienced developmental acclimation and/or transgenerational acclimation to projected future ocean conditions (e.g. elevated temperature and CO_2_).

### Inoculation of tolerant strains of microalgae endosymbionts in early life

Breeding programmes offer symbiotic microalgae to larval giant clams just before settlement, and the uptake of these symbionts are crucial for the survival of the larvae. Since giant clam offspring do not acquire their microalgae endosymbionts from their parents, larvae at a few days old gain their symbiotic microalgae from the environment independently. It is therefore plausible to help build resilience in giant clam holobionts by offering tolerant strains of microalgae symbionts at this stage.

In general, studies have found that giant clams in the Indo-Pacific region generally associate with three genera of Symbiodiniaceae: *Symbiodinium*, *Cladocopium* and *Durusdinium*, where each genus possesses unique ecological characteristics. For instance, *Symbiodinium* species are most adapted to living in shallow-water environments and tolerant to high irradiance stress (Venn *et al.*, 2008), while *Durusdinium* species are typically found in symbiosis with hosts living in stressful environments characterized by large diel or seasonal shifts in temperature and/or broad fluctuations in water turbidity (Tanzil *et al.*, 2016). Concurrently, depending on the giant clam species and/or environmental conditions, specific species of dinoflagellates may be chosen for inoculation to increase the survival of giant clam larvae and juveniles.

Among giant clam species, some are faster growing than others (e.g. *T*. *gigas* grows 8–12 cm.yr^−1^ versus *T*. *squamosa* that grows 2–4 cm.yr^−1^; Beckvar, 1981). Symbiont type can also influence growth. Fitt (1985) found that inoculation of fast-growing symbiont species to larvae and juveniles of *H*. *hippopus* led to higher growth and survival rates. Symbiont species also differ among giant clam species and with host size; smaller giant clams host a more diverse array of symbiont genera than larger clams (Ikeda *et al*., 2017). Indeed, the use of small, rather than medium-sized, giant clams to inoculate juvenile (seed) giant clams with symbionts increases survival two-fold, likely because of the increased Symbiodiniaceae diversity (Yamashita *et al*., 2021).

On the other hand, regional environmental conditions can also determine symbiont type in giant clams. Red Sea giant clams (*T*. *maxima* and *T*. *squamosa*) exclusively host *Symbiodinium* symbionts, suggesting that this specific host-endosymbiont interaction could be beneficial in the relatively extreme heat and irradiance conditions characteristic of the Red Sea (Pappas *et al.*, 2017). A greater understanding of giant clam host-endosymbiont interactions will therefore allow breeders to optimize the introduction of appropriate tolerant symbiont species to enhance survival of larvae and juveniles in mariculture. Such an approach may give young giant clams a head start in life and allow them to better cope under rapidly changing field conditions.

All the strategies above still require the reduction of global CO_2_ emissions and for nations to move to net zero emissions as soon as possible and potentially beyond net zero emissions. Continuing emissions on the current worst case RCP8.5 projection trajectory (Collins *et al*., 2013) will mean coastal oceans will not be conducive to giant clam survival in forthcoming decades. While breeding programmes can help restock certain areas and provide a supply to replace clam-depauperated areas, there are high operational and labour costs and substantial equipment and facilities involved. Restocking programmes are not the primary solution to overexploitation or global change, instead the challenge is to protect the remaining wild populations of giant clams and their coral reef habitats while global emissions, and thus environmental conditions, stabilize.

## Tourism potential of giant clams in a changing world

As charismatic megafauna, and one of the top iconic animals to see on coral reefs, giant clams hold significant potential for ecotourism, and tapping into the aesthetic value of giant clams as a tourist attraction can provide an additional conservation incentive. They may also possess a greater tolerance to some environmental change stressors as opposed to corals, meaning tourists could still view giant clams in areas where coral is degraded. In locations such as the Great Barrier Reef in Australia and Samal and Tawi-Tawi in the Philippines, giant clams are used in snorkel trails or in giant clam gardens for tourists. Giant clams are particularly amenable to tourist viewing as they (i) are sessile, unlike the majority of other iconic coral reef megafauna, (ii) inhabit shallow depths, providing good viewing opportunities on snorkel, (iii) are conspicuous, (iv) are long-lived, and (v) can be translocated into position. Aquacultured giant clams are well suited for translocation, ideally while small to medium sized. Giant clams can also be incorporated into citizen science programmes to enhance conservation efforts (e.g. Requilme *et al*., 2021). We suggest giant clams be flagship species (Soo and Todd, 2014; Neo *et al*., 2015) for coral reef habitats to help raise public awareness and whereby protection for other species, such as reef-building coral and coral reef inhabitants, would come with the protection of environmental conditions conducive to the survival of the charismatic ambassador species.

The Great Barrier Reef injects AUD$6.4bn (USD$5.0bn) into the Australian economy every year (Deloitte, 2017), and recognizing the monetary tourism value for threatened habitats and iconic species can serve as a strong conservation incentive in a currency that can be understood by policy makers and the general public. For example, manta rays are fished for their meat, but in Yap the lifetime value of each manta ray is estimated at ~USD$1.9 million because of the ecotourism revenue generated when tourists pay to watch manta rays (O’Malley *et al*., 2013). Demonstrating the enormous continuing revenue from the live animal compared to the dead animal empowers local people to make informed choices and incentivizes protecting iconic species. To date, no studies have yet valued giant clams in terms of tourism revenue, and this is potentially an area for further research, on the intersection of economics and conservation science, that could help to save wild stocks. Additional high value ecotourism programmes, where tourists are able to help hands-on with giant clam breeding and restocking activities may help offset some of the relatively high costs of these activities.

## Future directions

While we show how results from studies on giant clam physiology, morphology and behaviour can begin to inform conservation strategies, more work on global change stressors is required to understand the responses of giant clams to these pervasive changes in natural ecosystems. Elevated temperatures are already affecting giant clam wild populations. Further work on responses of individuals maintained at both elevated temperatures and simulated heat stress events could help determine any bleaching thresholds and other responses among different species to assist management and conservation efforts. The effects of ocean acidification on giant clams are still relatively little studied, and consideration of emerging threats, such as ocean deoxygenation, is an area for further work. Also, the interaction of global- and local–regional scale stressors, such as ocean warming and ocean darkening or pollution, need to be understood.

For all these types of environmental change drivers, it will be important to assess a variety of biological responses, including physiology, morphology and behaviour, over various life stages to determine any life history bottlenecks. Additionally, the responses of communities within the holobiont, particularly the giant clam–Symbiodiniaceae association, as well as the response of the holobiont as a whole, will be important in assessing the overall resilience and adaptive capacity of giant clams to rapid environmental change. Transcriptomics could help unravel which genes are expressed at different environmental conditions and whether individuals or species have the capacity to upregulate critical genes to help cope with stressful conditions. Ecological modelling of biological responses of giant clams using physical and chemical data could help determine how the ranges of threatened species and populations may change over coming decades. Determining the economic value of giant clams for coral reef tourism and restocking activities could help incentivize and enhance their protection. Another opportunity to inform the conservation of these threatened species is to evaluate community assemblages using other types of information such as species richness, functional traits and phylogeny to identify species based on their functional and historical contributions. Such studies may also incorporate data on biological responses to holistically capture the species’ ecological standing within ecosystems.

While new knowledge on the biological responses of threatened species is often urgently needed, particularly in respect to how they respond to rapid environmental change, research on threatened species can be challenging. Fortunately, giant clams can be successfully spawned in captivity, allowing experimental work to be undertaken on captive bred individuals, particularly in partnership with conservation breeding programmes. In interpreting results from captive bred individuals, one should consider that broodstock will be from a more limited genetic pool than wild stock, may be provisioned differently and may be acclimated to captive conditions, and that these factors could affect the responses of offspring in positive or negative ways to environmental drivers investigated.

## Conclusion

Enhanced global efforts are urgently needed to meet net zero CO_2_ emissions as soon as possible and progress to beyond net zero CO_2_ emissions to give biodiversity the chance to survive the already locked-in changes to the Earth’s climate. Doing so will help to avoid lethal and sub-lethal effects of global change on threatened species, such as giant clams. In the meantime, this perspective article has (i) identified new environmental threats to giant clams, (ii) identified conservation management actions in the face of rapid environmental change, (iii) highlighted the importance of tourism in conserving giant clams and (iv) identified areas of further research on giant clams. While these actions are likely to allow the survival and appreciation of giant clams in the first half of this century, like coral reefs, their continued persistence in the world’s tropical oceans is ultimately dependent on rapid mitigation of global climate change.
